# Intelligent Diagnosis Based on Double-Optimized Artificial Hydrocarbon Networks for Mechanical Faults of In-Wheel Motor

**DOI:** 10.3390/s22166316

**Published:** 2022-08-22

**Authors:** Hongtao Xue, Ziwei Song, Meng Wu, Ning Sun, Huaqing Wang

**Affiliations:** 1 School of Automotive and Traffic Engineering, Jiangsu University, Zhenjiang 212013, China; 2 Bosch Automotive Products (Suzhou) Co., Ltd., Suzhou 215021, China; 3 College of Automotive and Traffic Engineering, Nanjing Forestry University, Nanjing 210037, China; 4 College of Mechanical and Electrical Engineering, Beijing University of Chemical Technology, Beijing 100029, China

**Keywords:** intelligent diagnosis, in-wheel motor, artificial hydrocarbon networks, K-means clustering, AdaBoost algorithm

## Abstract

To avoid the potential safety hazards of electric vehicles caused by the mechanical fault deterioration of the in-wheel motor (IWM), this paper proposes an intelligent diagnosis based on double-optimized artificial hydrocarbon networks (AHNs) to identify the mechanical faults of IWM, which employs a K-means clustering and AdaBoost algorithm to solve the lower accuracy and poorer stability of traditional AHNs. Firstly, K-means clustering is used to improve the interval updating method of any adjacent AHNs molecules, and then simplify the complexity of the AHNs model. Secondly, the AdaBoost algorithm is utilized to adaptively distribute the weights for multiple weak models, then reconstitute the network structure of the AHNs. Finally, double-optimized AHNs are used to build an intelligent diagnosis system, where two cases of bearing datasets from Paderborn University and a self-made IWM test stand are processed to validate the better performance of the proposed method, especially in multiple rotating speeds and the load conditions of the IWM. The double-optimized AHNs provide a higher accuracy for identifying the mechanical faults of the IWM than the traditional AHNs, K-means-based AHNs (K-AHNs), support vector machine (SVM), and particle swarm optimization-based SVM (PSO-SVM).

## 1. Introduction

With the outbreak of the oil crisis, many policies and measures have been introduced to promote the development of the new energy vehicle (NEV) from countries all over the world. As a result, many new driving technologies have become research hotspots, one of which is the in-wheel motor (IWM), which has the advantages of high efficiency, fast response, and full-time wire control. The IWM-based driving system can reduce vehicle energy consumption, improve vehicle performance, and optimize spatial layout. Therefore, the IWM-based driving system has been recognized as the ideal configuration of future NEV power systems [1,2]. However, the unique installation between the IWM and the suspension must increase the unsprung mass of a vehicle, whereby the vibration isolation performance of the suspension is deteriorated, and the operational stability and safety are compromised. Moreover, as each IWM must directly withstand the intermittent strong impact load from the road, its local structure, such as the bearing, can be broken very easily, and it is difficult to detect the subtle damage. At present, there is no system for monitoring the IWM’s condition. Once a fault occurs to one or more of the IWMs in the driving system, local driving performance should deteriorate, which creates a security threat for the safe operation of the whole vehicle [3,4]. Therefore, it is urgent to explore efficient and reliable diagnosis methods for monitoring IWM’s fault condition [5,6,7].

In recent years, studies have been performed on signal processing, feature extraction, intelligent diagnosis, and the condition recognition of the common motor. For example, an intelligent fault diagnosis algorithm has been proposed by adaptive transfer affinity propagation clustering, which can extract potential energy features from the intrinsic mode functions of vibration signals using complete ensemble empirical mode decomposition (EMD) with adaptive noise [8]. A dual-tree complex wavelet transform (WT) is employed to acquire the multiscale signal’s features, which improve the classification accuracy of a fault’s characteristic signal [9]. A novel intelligent fault diagnosis approach based on principal component analysis (PCA) and a deep belief network (DBN) has been presented to extract the fault signatures in terms of primary eigenvalues and eigenvectors [10]. Ensemble EMD, wavelet packet transform (WPT), and sparse representation (SR) are utilized to accurately extract the information of fault features which are buried in vibration signals [11,12]. The method of attribute selection and feature extraction based on random forest (RF) combined with PCA has a faster recognition time and a higher recognition accuracy than other algorithms [13]. The integrated time-domain, frequency-domain statistical characteristics, EMD, and deep learning methods are proposed to realize the automatic recognition of different fault states of rotating machinery [14,15]. These methods of signal processing and feature extraction have the advantages of high resolution and strong interference suppression ability.

Moreover, Lagrange particle swarm optimization (L-PSO) has been improved to establish the multiple fault diagnosis model, which verifies the effectiveness and stability by sensor data-based multiple fault diagnosis [16]. Support vector machines (SVMs) have been applied to report the health states of railway turnout with high accuracy and self-adaptability [17,18]. A neural network (NN) is used establish the fault diagnosis model of large-scale ship engines, which has a higher diagnostic accuracy and use value [19,20]. The convolutional NN is modified with transfer learning for analyzing the thermal images of the rotor-bearing system under different working conditions [21]. The ensemble deep auto-encoders (EDAEs) method is proposed to intelligently diagnose the faults of rolling bearings [22]. A novel model with continuous WT and a local binary convolutional NN is applied to intelligently diagnose the faults of rotating machinery [23,24,25]. An improved particle swarm optimization variational mode decomposition (IPVMD) and improved convolutional neural network (I-CNN) are proposed to solve the problem of planetary gearbox composite fault diagnosis [26]. The improved convolutional neural network–support vector machine (CNN-SVM) method is presented to extract representative features from the multichannel vibration signals of the rolling bearing [27]. A novel tracking deep wavelet auto-encoder (TDWAE) method is introduced for the intelligent fault diagnosis of electric locomotive bearings [28]. The diagnosis method based on the auto-encoder and extreme learning machines is proposed for diagnosing faults in bearings so to overcome the deficiencies of longer training times [29]. A new real-time diagnosis method based on the dynamic Bayesian network (DBN) is used to distinguish the IWM’s mechanical faults [30]. The above methods have good classification effects or better applicability for specific application scenarios, but it is essential to establish the operation classification model and summarize the algorithm that can play better roles for specific scenarios [31,32,33]. However, as IWMs are often working in variable and complex conditions, the methods directly applied for fault diagnosis show neither superior performance nor acclimatization [34,35]. Therefore, it is urgent to make some improvement and achievement with respect to the existing artificial intelligence technologies so to establish the optimal intelligent diagnosis model for IWMs [36].

Artificial hydrocarbon networks (AHNs) are a new artificial intelligence algorithm that have excellent information encapsulation and integration ability. AHNs can not only realize the classifier function by using the target information in adjacent molecules, but also have the advantages of a clear network topology and strong adaptability [37]. However, while better performance would be achieved by training large samples, AHNs are confronted with large computational quantities and response times in these application [38,39,40].

To solve the above problems, K-means clustering and the AdaBoost algorithm are employed to design double-optimized AHNs with the core idea to improve the interval updating method of any adjacent AHN’s molecules and the linear connection scheme of multiple molecules. Moreover, the iterative process and computing speed of the proposed methods are researched, and the classification accuracies are also compared with several existing methods under different operating conditions. The rest of the paper is organized as follows. The basic theory of traditional AHNs is introduced in Section 2, and the improved method of the double-optimized AHN algorithm is presented in Section 3. Experimental results and analysis are mainly described in Section 4. Conclusions are summarized and future research is determined in Section 5.

## 2. Artificial Hydrocarbon Networks

Artificial hydrocarbon networks (AHNs) are a new paradigm of computational algorithms whose framework is a chemically inspired technique based on organic chemistry, and many of the technical terms, such as atom, CH molecule, compound, and mixture, inherit from hydrocarbon networks. Therefore, many properties of organic chemistry become the main characteristics of the AHNs’ algorithm, such as structural organization, clustering information, inheritance of behavior, encapsulation of data, and stability in structure and response. There are some studies on the application of AHNs in the fields of signal processing and condition recognition [39,40].

**Figure 1:**
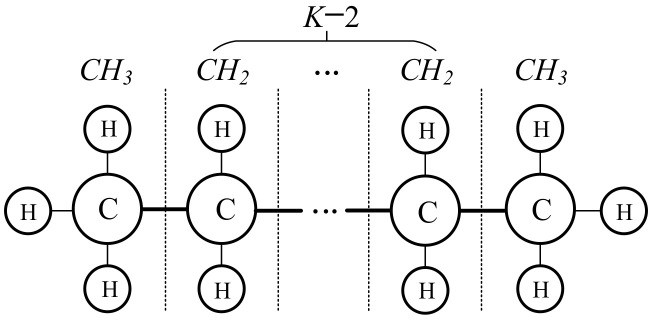
Common graph structure of an AHN.

In general, a linear and saturated chain of hydrocarbons, as shown in Figure 1, is used to establish the graph structure of an AHN that represents their physical properties, and the chemical behaviors of the components and their interactions are modeled through a mathematical object that can describe the nonlinear relationships between the input (attribute) and the output (target) variables. Certainly, these variables can be a number or vector. For convenience, let *x* (*x* ∈ [*a*, *b*]) be any chemical environment, *f*(*x*) be the corresponding chemical behaviors, and a mathematical model of an AHN can be described by

(1) $$y=f(x)=\sum_{k=1}^{K}C_{k}\prod_{r}\left(x-h_{kr}\right)\begin{array}{cc} & x\in [a,\;b] \end{array}$$

where *K* is the number of CH primitive molecules in the AHNs, *C_k_* is the carbon atomic value in the *k*-th hydrocarbon molecule, and *h_kr_* is the *r*-th hydrogen atomic value around the carbon atomic *C_k_*. According to chemical rules, *r* is a positive integer up to 4.

When two or more AHNs are mixed together, the resultant mixture contains more information. Suppose any number of AHNs can interact without sharing electrons, the optimal ratios of AHNs can be found to obtain the minimum loss energy in the whole structure. Then, the whole model of an AHNs can be defined by

(2) $$Y=\sum_{j=1}^{J}\alpha_{j}\cdot y_{j}$$

where *y_j_* is the output value of the *j*-th AHN model, *α_j_* is the stoichiometric coefficient of the *j*-th AHN model, and *J* is the number of AHNs in a system.

In practical application, the number of hydrocarbons *J* and the number of each hydrocarbon molecule *K* are determined according to practical engineering problems. When AHNs are used to build the system of condition recognition, *J* depends on the engineering requirement and *K* hinges on the output class. Usually, it is easier to determine the values of *J* and *K* for specific classification, but harder to find the optimal values of carbon atomic *C_k_* and hydrogen atomic *h_kr_*. This is because the input domain *D_k_* excites the *k*-th molecule. For the input domain *D_k_*, ${⋃}_{k=1}^{K}D_{k}=\left[a,\;b\right]$, $D_{k}\;⋂\;D_{n}=\emptyset \;\left(k\;\neq \;n\right)$, and the initial domain, *D_k_*^(0)^ is assigned by the equipartition method [34]; then, the criterion of minimizing the absolute errors is employed to determine the attribution domain of each sample. Suppose Σ = {(*x*, *y*)|*x* ∈ *X*, *y* ∈ *Y*} to be a training set, and *M* to be the number of training samples. Each sample (*x_m_*, *y_m_*) (*m* = 1, 2, …, *M*) includes the state parameter and the state label. Then, the attribution domain *z_k_* of each sample (*x_m_*, *y_m_*) can be expressed as follows:

(3) $$z_{k}=\arg\underset{k}{\min}|x_{m}-(2k-1)*\frac{b-a}{2K}|$$

In this way, all training samples are assigned to the different input domains, then the attribution set *Z* = {*z*_1_, *z*_2_, …, *z_k_*, …, *z_K_*} is obtained to activate the parameters of the AHN’s model. In this traditional approach, the least square method is used to determine the values *C_k_*, *h_kr_* of the carbon atom, and the hydrogen atom in each hydrocarbon molecule, and an AHN’s model can be expressed as follows:

(4) $$\Lambda =\{C_{1},\cdots ,\;C_{K};\;h_{11},\cdots ,\;h_{1r},\cdots ,\;h_{Kr};\;D_{1},\cdots ,\;D_{K}\}$$

Similarly, the different training sets are used to build the corresponding AHN’s models. However, it is difficult to evaluate the stoichiometric coefficient of each AHN model. At present, the classification abilities of different training sets are compared, and the weight matching method is employed to confirm the coefficient *α_j_*, then multiple AHN models are synthesized into a complete classifier called AHNs.

**Figure 2:**
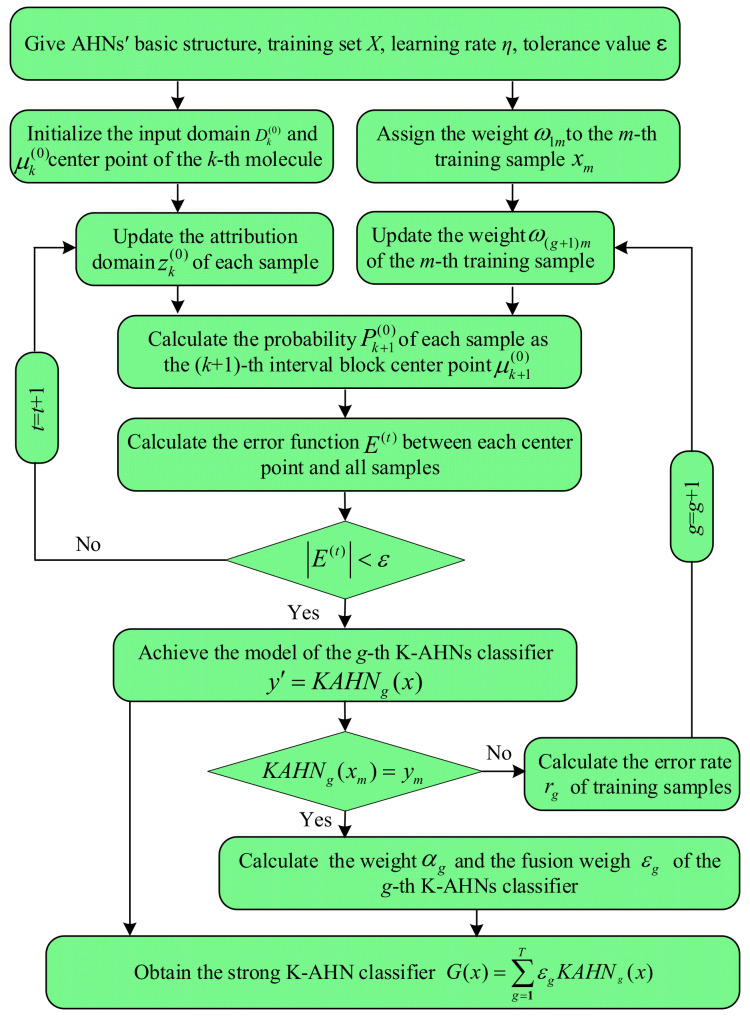
Flow chart of double-optimized AHNs.

In fact, there are two problems to confront in the renewal of the hydrocarbon molecular interval by the traditional AHN method: the single way and the slow convergence speed. Therefore, it is difficult for the traditional AHN method to be competent in a complex environment and attain the rapid response requirement. Based on this, K-means clustering and AdaBoost algorithms are employed to improve the clustering rule of the hydrocarbon molecular interval and linear connection scheme for hydrocarbons, as well as perfect the multistate classification model. Since the traditional AHN algorithm is optimized twice, the improved AHN is called the “double-optimized AHNs” in the paper. Figure 2 is the flow chart of double-optimized AHNs.

## 3. Double-Optimized AHNs

### 3.1. AHNs’ Model Optimization Based on K-Means

The K-means algorithm is a common clustering method which uses Euclidean distance to attribute the samples with a high similarity and a low difference to the same interval so as to form multiple interval blocks [41,42]. To make the distance between the center point as large as possible so to accelerate the convergence speed and improve the classification error in the subsequent iteration processing when the AHNs model is trained, the K-means algorithm is applied to optimize the update mode of the hydrocarbon molecular interval, and then improve the training speed of the AHNs model and reduce the strict requirements of the training sample set.

In the initialization process of the AHNs’ hydrocarbon molecular interval, the number of hydrocarbon molecules *K* is regarded as the number of clustering intervals, and the input sample set *X* and the center point of each hydrocarbon molecular interval block are made to classify the input activation parameters *Z* corresponding to different hydrocarbon molecular interval blocks.

In general, the number of K-means clusters is defined as the number of hydrocarbon molecules *K* of the AHN model, and a sample is selected randomly as the center point of the first interval block in the interval $\mu_{1}^{\left(0\right)}$ from the input sample set *X*. Secondly, the Euclidean distance between the remaining input samples *x_m_* and the center point of the existing interval block is calculated to obtain the probability *P* that each sample is selected as the center point of the next interval block, and then the input sample with the highest probability is selected as the center point of the next interval block, and so on until the initialization of *K* interval block centers is completed. Let $P_{k\;+\;1}^{\left(0\right)}\left(x\right)$ be the probability of an input sample *x* is selected as the (*k +* 1)-th interval block center point $\mu_{k\;+\;1}^{\left(0\right)}$ in the initialization processing, then $P_{k\;+\;1}^{\left(0\right)}\left(x\right)$ and $\mu_{k\;+\;1}^{\left(0\right)}$ are expressed as follows:

(5) $$P_{k+1}^{\left(0\right)}(x)=\frac{R(x,\mu_{k}^{\left(0\right)})}{\sum_{m=1}^{M}R(x_{m},\mu_{k}^{\left(0\right)})}$$

(6) $$\mu_{k+1}^{\left(0\right)}=\arg\underset{x\in X}{\max}P_{k+1}^{\left(0\right)}(x)$$

where $\mu_{k}^{\left(0\right)}$ is the *k*-th interval block center point in the initialization process, $R\left(\cdot \right)$ is the Euclidean distance, and *M* is the number of training samples. In that way, the *K* interval block center points have been obtained to complete the initialization of center point set $U^{\left(0\right)}$ of hydrocarbon molecule interval block, as follows:

(7) $$U^{\left(0\right)}=\{\mu_{1}^{\left(0\right)},\;\mu_{2}^{\left(0\right)},\;\cdots ,\;\mu_{k}^{\left(0\right)},\;\cdots ,\;\mu_{K}^{\left(0\right)}\}$$

In the following process, the interval block center point set $U^{\left(t\right)}$ in the *t*-th $\left(t\;\geq \;1\right)$ clustering process is used to find the interval block center point with the shortest Euclidean distance, then the input sample *x_m_* is classified into the corresponding interval blocks for obtaining the input activation parameter set *Z* corresponding to *K* interval blocks, in which the input activation parameter $z_{k}^{\left(t\right)}$ corresponding to the *k-*th interval block in the *t-*th clustering process can be expressed as follows:

(8) $$z_{k}^{\left(t\right)}=\arg\underset{x_{m}\in X}{\min}R(x_{m},\mu_{k}^{\left(t\right)})\mu_{k}^{\left(t\right)}\in U^{\left(t\right)}$$

The above algorithm is performed to satisfy the convergence condition of the improved AHN model. When the algorithm is iterating, the input activation parameters $z_{k}^{\left(t\right)}$ of each interval block is used to calculate the corresponding center point. Let $E^{\left(t\right)}$ be the error function of the improved AHN model in the *t-*th clustering process, where it is judged whether $E^{\left(t\right)}$ reaches the convergence condition. If the convergence condition is met, the training samples have been classified into the different hydrocarbon molecular interval. Otherwise, the *k*-th interval block center point $\mu_{k}^{\left(t\;+\;1\right)}$ in the (*k +* 1)-th clustering process is updated by the sum of the Euclidean distances between each center point and all samples in $z_{k}^{\left(t\right)}$. The center point corresponding to the smallest one is confirmed as $\mu_{k}^{\left(t\;+\;1\right)}$, as follows:

(9) $$E^{\left(t\right)}=\sum_{k=1}^{K}\sum_{x\in z_{k}^{\left(t\right)}}R(x,\mu_{k}^{\left(t\right)})$$

(10) $$\mu_{k}^{\left(t+1\right)}=\arg\underset{\lambda }{\min}\sum_{x\in z_{k}^{\left(t\right)}}R(x,\lambda )$$

Then, the center point set $U^{\left(t\right)}$ in the *t-*th clustering process is used to drive each hydrocarbon molecule interval block $D^{\left(t\right)}$, as follows:

(11) $$D^{\left(t\right)}=\left\{\begin{array}{l} {}[a,\;(\mu_{1}^{\left(t\right)}+\mu_{2}^{\left(t\right)})/2),\;\;\;\;\;i=1\\ {}[(\mu_{i-1}^{\left(t\right)}+\mu_{i}^{\left(t\right)})/2,(\mu_{i}^{\left(t\right)}+\mu_{i+1}^{\left(t\right)})/2),\;i=2,\cdots ,K-1\\ {}[(\mu_{K-1}^{\left(t\right)}+\mu_{K}^{\left(t\right)})/2,\;b],\;\;\;\;\;i=K \end{array}\right.$$

Finally, the least square method is employed to determine new AHN model, such as *C_k_*, *h_kr_*, and *D_k_*. This has completed the first optimization of the AHN model based on the K-means algorithm. In this paper, the improved AHN model based on K-means is denoted as the K-AHNs model, and the output of the K-AHNs model is expressed as follows:

(12) $$y^{\prime }=KAHN(x)$$

### 3.2. K-AHNs’ Model Optimization Based on AdaBoost

Adaptive boosting (AdaBoost) is a familiar iterative algorithm, where the core idea is to train different classifiers for the same training data and then combine these classifiers to form a stronger classifier [43,44]. In the process of algorithm improvement, each classifier will use an adaptive resampling technique to choose different samples, whereby the misclassified samples produced by previous classifiers are focused to form a new training sample with the other data. Moreover, the misclassified samples are endowed with higher weights to train the next classifier. The final classifier is a weighted sum of the ensemble predictions. Therefore, the AdaBoost algorithm is often applied to solve two-class problems, multiclass single-label problems, multiclass multilabel problems, categories of single-label problems, and regression problems [45].

In this paper, the AdaBoost algorithm is used to optimize the K-AHNs model for solving the strong dependence on the distribution of training samples. For the various samples, the sensitivities of these classifiers are made to assign the optimal weights for weak K-AHNs, then weaken the weight of the weak classifier and enhance the evaluation grade of stable one in the linear combination of all classifiers. In general, it takes four steps to achieve the optimization process of the K-AHNs model based on AdaBoost, as follows:

Step 1: Assign a weight to each training sample and obtain the first K-AHNs classifier. In the initial process, each example is endowed with the same weight. Suppose there are *M* samples in the training set *X*, the weight *w*_1*m*_ of each sample *x_m_* (*x_m_* ∈ *X*, *m* = 1, 2, …, *M*) is set as 1/M, then all samples with the same weights are trained to obtain the first K-AHNs classifier. Usually, the classifier is weak.

Step 2: Calculate the error rate of the training samples and determine the weight of the corresponding classifier. The training result of the first K-AHNs classifier is analyzed, especially the misclassified samples, and the error rate *r_g_* of the training samples in the *g*-th K-AHNs classifier is defined as follows:

(13) $$r_{g}=P(KAHN_{{}^{g}}(X)\neq Y)=\sum_{m=1}^{M}w_{gm}h(KAHN_{{}^{g}}(x_{m})\neq y_{m})$$

(14) $$h(KAHN_{{}^{g}}(x_{m}),y_{m})=\left\{\begin{array}{c} 1,KAHN_{{}^{g}}(x_{m})\neq y_{m}\\ 0,KAHN_{{}^{g}}(x_{m})=y_{m} \end{array}\right.$$

where *KAHN_g_*(*·*) is the output result of the *g*-th K-AHNs classifier and *w_gm_* is the weight of *m*-th sample in the *g*-th K-AHNs classifier. While the error rate *r_g_* is determined, the weight *α_g_* of the *g*-th K-AHNs classifier can be calculated for comprehensive evaluation, as follows:

(15) $$\alpha_{g}=\frac{1}{2}ln(\frac{1-r_{g}}{r_{g}})$$

where *ln*(*·*) is the natural logarithm function.

Step 3: Update the weights of each sample and K-AHN classifier in next process. According to the weight of each sample *w_gm_* and the K-AHN classifier *α_g_* in the *g*-th process, the weight of each sample *w*_(*g* + 1) *m*_ in the (*g* + 1)-th process can be updated as follows:

(16) $$w_{(g+1)m}=\frac{w_{gm}}{Z_{g}}\exp[\alpha_{g}\cdot (KAHN_{g}(x_{m})-y_{m})]$$

where *Z_g_* is the normalization factor of the *g*-th K-AHN classifier. Based on this, the weights of the misclassified samples will be increased progressively in exponent regularity.

Step 4: Obtain the strong K-AHN classifier. When the above operation is repeated *T* times, these weak classifiers are weighted to fuse into a strong classifier *G*(*x*), as follows:

(17) $$G(x)=\sum_{g=1}^{T}\varepsilon_{g}KAHN_{{}^{g}}(x)$$

where $\varepsilon_{g}$ is the fusion weigh of the *g*-th K-AHN classifier in the final classifier, $\varepsilon_{g}=\alpha_{g}/\sum_{g=1}^{T}\alpha_{g}$.

## 4. Experimental Verification

To verify the effectiveness of double-optimized AHNs for diagnosing some mechanical faults, two cases of bearing data are studied, including Case 1: the bearing data from Paderborn University, and Case 2: the bearing data from the self-made IWM test stand. The experimental data of Case 1 are representative in the field of fault diagnosis, where traditional AHNs, K-AHNs, and double-optimized AHNs will be used with the same training data and test data for comparison and discussion. However, the experimental data of Case 2 are especially particular in the application scenarios, where the robustness and diagnosis accuracy of double-optimized AHNs can be compared with the existing methods.

### 4.1. Case 1: The Bearing Data from Paderborn University

The experimental data of the bearing faults from Paderborn University [46] are firstly analyzed. Single faults with inner race and outer race defects were set on the testing bearing (ball bearing with Type 6203) separately, and the extent of the bearing defect was cut by an electric engraver into a trench of a 0.25 mm length in the rolling direction and a depth of 1–2 mm, respectively. The operating condition was that the rotational speed was 900, 1500 rpm, the load torque was 0.1, 0.7 Nm, and the radial force was 1000 N. The vibration was measured with the sampling frequency of 64 kHz.

The vibration signal in each condition was firstly processed by empirical wavelet transform (EWT) [47] to extract five highly sensitive symptom parameters (SPs), such as the root mean square (RMS), average peak, skewness, kurtosis, and waveform stability index, wherein the SPs are labeled with SP_1_, SP_2_, SP_3_, SP_4_, SP_5_ [37,48], respectively. Then, the five-dimensional vector, namely (SP_1_, SP_2_, SP_3_, SP_4_, SP_5_), is used to represent the bearing state within a certain time. In this paper, the five states of the bearing from Paderborn University are selected, including the normal state (State 1), the slight fault of inner ring (State 2), the severe fault of inner ring (State 3), the slight fault of outer ring (State 4), and the severe fault of outer ring (State 5). The vibration data of each state every 0.128 s are regarded as a sample to set s vector of SPs, then 30 samples are obtained in each state of bearing.

**Figure 3:**
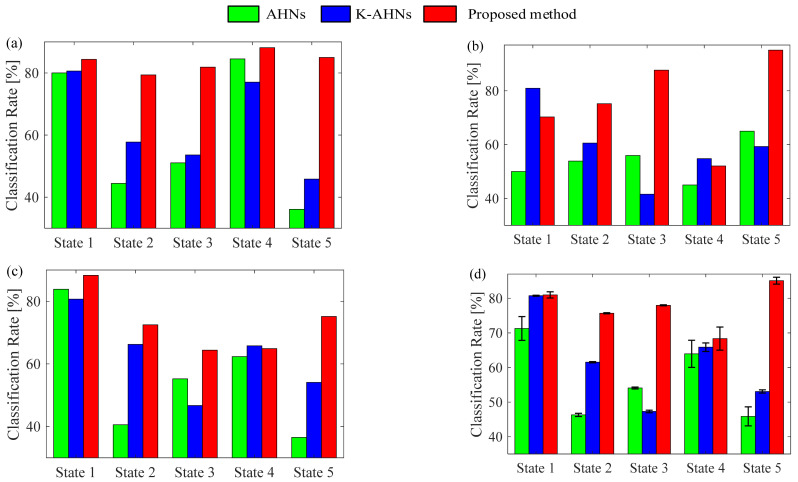
The classification results of the five states of the motor bearing of Paderborn University under three operating conditions (**a**) Rotational speed = 1500 rpm, load torque = 0.7 Nm; (**b**) Rotational speed = 900 rpm, load torque = 0.7 Nm; (**c**) Rotational speed = 1500 rpm, load torque = 0.1 Nm; (**d**) Average classification rate and error degree.

According to the actual condition of the above bearing experiment, the double-optimized AHNs model is built with a five-type classifier. The input *x_m_* is five-dimensional vector (SP_1*m*_, SP_2*m*_, SP_3*m*_, SP_4*m*_, SP_5*m*_), and the corresponding output is an integer of 1, 2, 3, 4, or 5 which maps into the state with the same number. Then, 70 percent of each of the state samples are randomly selected to associate with the corresponding label for the training samples {(SP_1_, SP_2_, SP_3_, SP_4_, SP_5_, *y*) | *y* = 1, 2, 3, 4, 5}, while the other samples are used to test the performance of the double-optimized AHNs classifier. Certainly, the basic parameters of each classifier are set in advance. In particular, the learning rate is 0.1, the tolerance value is 0.05, and the maximum number of iterations is 50. Therefore, the diagnosis system based on the double-optimized AHNs is performed. When the output value agrees with the defined label, it indexes the stipulated state, and the diagnosis result is correct. Otherwise, the state is a case of mistaken identity. Similarly, the diagnosis models based on the AHNs and K-AHNs are performed with the same training and test samples, respectively. Then, the diagnosis accuracies of all the test samples of the different bearing states under the three operating conditions are shown in Figure 3. Moreover, the average CPU times of the three methods in the training and test processes are shown as Table 1.

Obviously, there are great differences in the classification accuracies of three classifiers. In the first condition, with a rotational speed of 1500 rpm and load torque of 0.7 Nm, the diagnosis accuracies of the double-optimized AHNs proposed in this paper are all over 80%, and the classification results of the AHNs and K-AHNs are in fluctuation, especially the accuracies that are less than 60% for identifying State 2, State 3, and State 5. In the other operating conditions, the performances of these methods show a drop, but nearly all accuracies of the proposed method are still higher than 60%, and almost half of the results based on AHNs and K-AHNs are less than 60%. Using statistical analysis, the average accuracy of the proposed method is 7.35% more than the K-AHNs, and 11.99 % more than the AHNs. Moreover, the variance error of the proposed method is less than 0.39%. As far as the average CPU times are concerned, the training and test processes are discussed separately. In the training process, the processing rate of the proposed method is obviously faster than the traditional AHNs but slower than the K-AHNs. In the test process, the processing speed of the proposed method is well up to the leading level. On the whole, the performance of the proposed method is the highest, followed by the K-AHNs model.

### 4.2. Case 2: The Bearing Data from Self-Made IWM Test Stand

**Figure 4:**
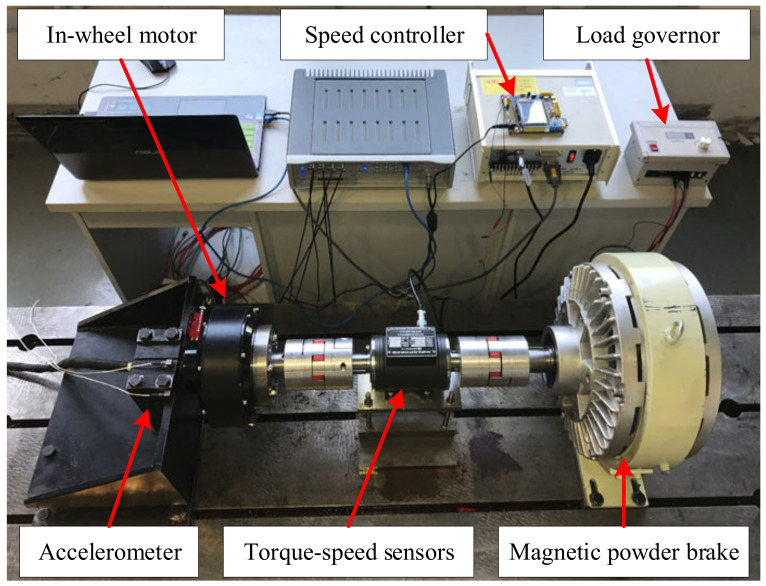
The test bench system of IWM.

**Figure 5:**
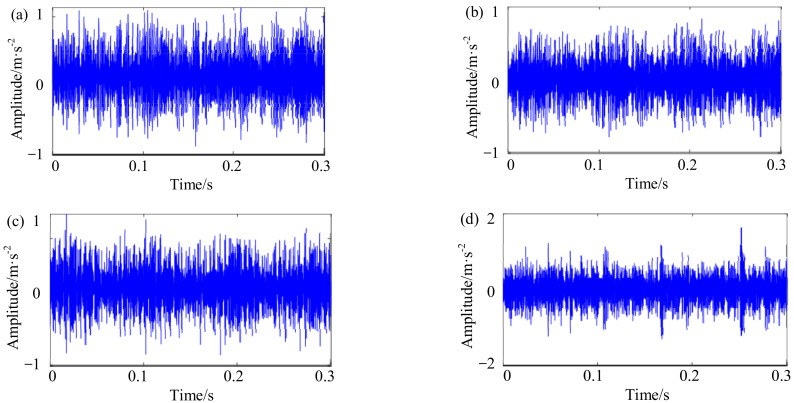
Vibration signals of four IWMs at the rotating speed of 700 rpm and load torque of 30 N·m: (**a**) State 1—normal state; (**b**) State 2—rolling element defect; (**c**) State 3—inner ring defect; (**d**) State 4—outer ring defect.

Figure 4 shows the self-made IWM test bench, which is mainly composed of batteries, the IWM, an inverter, and a magnetic powder brake. Since the bearing defect is a typical machine fault of IWMs, double-rowed tapered rolling bearings (Type: DU2505237) are artificially processed with a single damage (width 0.5 mm and depth 0.15 mm) across rolling element, inner ring, and outer ring, which are then fixed tightly on the stator axis of each IWM by a professional, respectively. In the process of the experiment, four IWMs are orderly operated with the same speed and load, including the normal state (State 1), the rolling element defect (State 2), the inner ring defect (State 3), and the outer ring defect (State 4). Moreover, the operating condition of each state is relatively fixed in different ways. For example, the single chip microcomputer is used to simulate the gas pedals of the NEV for controlling each IWM at the rotating speeds of 100, 200, …, 700 rpm (or thereabouts). The magnetic powder brake is adjusted by the tension controller to the different loads of 0, 10, 20, and 30 N m. The vibration from the stator axis of each IWM is collected with a sampling frequency of 12.8 kHz to last for 60 s. Figure 5 shows the part of the vibration signals of four IWMs at the rotating speed of 700 rpm and load torque of 30 N m.

**Figure 6:**
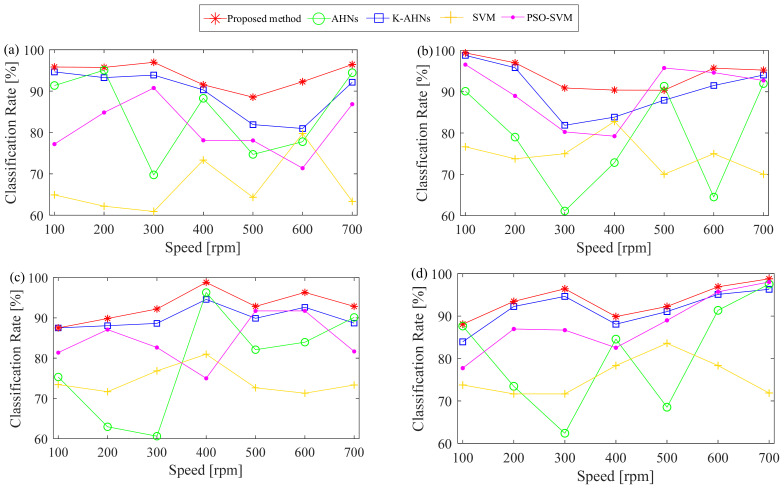
The classification results of five methods under different operating conditions. (**a**) No-load condition, (**b**) 10 N·m load condition, (**c**) 20 N·m load condition, (**d**) 30 N·m load condition.

All the raw signals are filtered with a 1–5 kHz band-pass filter. Since the fault features of the IWM at the lowest rotating speed are analyzed, the vibration data every 1.28 s are regarded as a sample to calculate the same SPs above, and then 45 samples of each IWM’s state are obtained in each operating condition. Based on the four states of the IWMs, the double-optimized AHNs model is set to a four-type classifier. The input *x_m_* is still a five-dimensional vector (SP_1*m*_, SP_2*m*_, SP_3*m*_, SP_4*m*_, SP_5*m*_), and the corresponding output becomes an integer of 1, 2, 3, or 4, which maps into the IWM’s state defined by the same number. Then, 70 percent of each state samples are randomly selected to associate with the corresponding label for the training samples {(SP_1_, SP_2_, SP_3_, SP_4_, SP_5_, *y*) | *y* = 1, 2, 3, 4}, while other samples are used to test the performance of the double-optimized AHNs classifier. Moreover, the learning rate is 0.1, the tolerance value is 0.05, and the maximum number of iterations is 50. Then, the double-optimized AHNs classifier is firstly performed and the classification results of four IWM’s states are computed in the same operating condition. For example, all test samples of the four IWMs at the rotating speed of 100 rpm and the no-load condition, that is, the load of 0 N m, are regarded as the different states of an IWM, and the IWM’s state is correctly judged as long as the output value agrees with the defined label. Then, the classification results are shown in Figure 6.

To reveal the effectiveness of the proposed method, four existing classifiers, the AHNs, K-AHNs, SVM [49], and the particle swarm optimization-based SVM (PSO-SVM), [50] are employed to build the diagnosis system. Here, the basic parameters of the AHNs and the K-AHNs are set as double-optimized AHNs. In this paper, two methods of the SVM and the PSO-SVM directly select the same configuration parameters of the relevant references to establish the classification systems, respectively. For example, Ref. [49] used the SVM with the Gaussian RBF function and the width parameter of 0.4, while Ref. [50] set the parameters of the PSO algorithm, such as two acceleration factors of 1.5, weight coefficient of 1, maximum iterations of 40, and population size of 20, to determine automatically the two key parameters of the SVM. Moreover, the same training samples above were used to train the classifier based on each method, then the same test samples were regarded as an unknown state to verify the classification performance of each method. The corresponding diagnosis results are shown in Figure 6.

**Figure 7:**
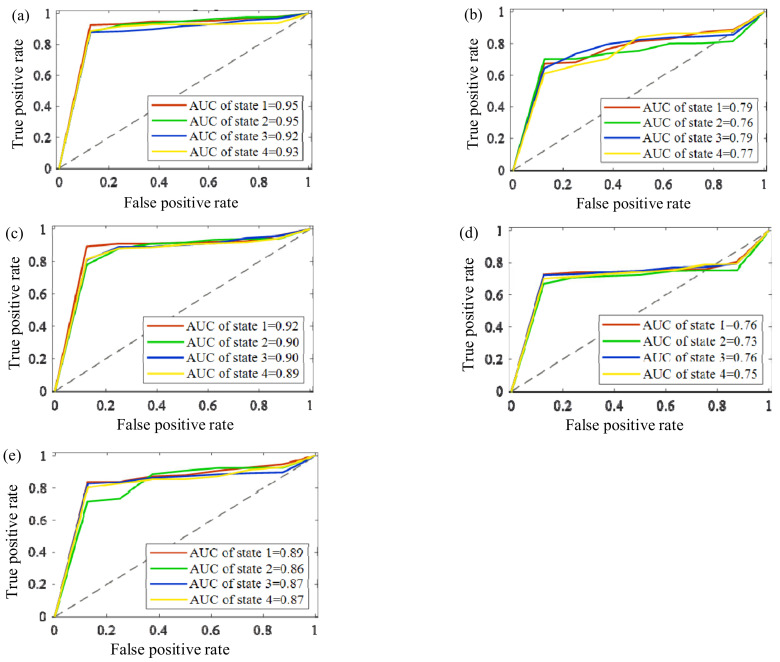
The ROCs and AUCs of the bearing states from the self-made IWM test stand by five methods: (**a**) proposed method, (**b**) AHNs, (**c**) K-AHNs, (**d**) SVM, (**e**) PSO-SVM.

Since the receiver operating characteristic (ROC) curve and the area under the curve (AUC) have a better sensitivity and specificity evaluation standard for each fault state, the ROC and AUC are used to judge the quality of the above classifiers. To better eliminate the contingency of the experiment and reflect the sensitivity and specificity of different operating states, seven random trials were conducted to show the ROCs and AUCs of bearing states from the self-made IWM test stand by the five methods shown in Figure 7.

**Figure 8:**
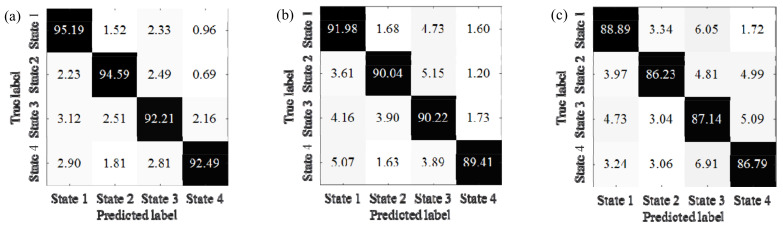
The confusion matrixes among IWM’s bearing states by different methods: (**a**) proposed method, (**b**) K-AHNs, (**c**) PSO-SVM.

Obviously, the proposed method, K-AHNs and PSO-SVM, have better performances. Hence, their specific confusion matrixes among the IWM’s bearing states are further analyzed, as shown in Figure 8.

It is clear that the double-optimized AHNs method has a better identification capability and robustness thanks to the classification accuracies of more than 88%, with the AUCs and diagnosis accuracies of the IWM’s bearing states being over 92% no matter how the rotating speed and load condition change. The second is the K-AHNs method, which maintains a high classification accuracy above 80%, where the AUCs and diagnosis accuracies of the IWM’s bearing states are over 89% under four different operating conditions. The condition recognition level based on the PSO-SVM algorithm can hover around 85%, but the stability is not better than the K-AHNs. The traditional methods of the AHNs and SVM cannot meet the engineering requirements in the fault diagnosis of the IWM with a variable working condition.

## 5. Conclusions

To effectively identify the mechanical faults of the IWM under variable rotating speeds and load conditions, double-optimized AHNs are proposed to build an intelligent diagnosis system, and the effectiveness is experimentally verified by two case studies of using datasets from Paderborn University and a self-made IWM test stand. The superiority of the method proposed in this paper can be summarized by the following points:

(1) K-means clustering and AdaBoost are used to optimize the AHNs algorithm, which not only simplify the complexity of the AHNs model, but also reconstitute the network structure of the AHNs; as a result, the double-optimized AHNs displays excellent performance due to the organic fusion of AHNs, K-means clustering, and AdaBoost mainly.
(2) As long as the intelligent diagnosis system is built by the double-optimized AHNs, no matter how the rotating speed and load conditions of the IWM are altered, the high classification accuracy can be obtained. It is attributed primarily to the strong robustness of double-optimized AHNs.
(3) The intelligent diagnosis method based on the double-optimized AHNs can avoid selecting configuration parameters and adaptively distribute the weight of multiple weak models for a strong classifier.

This paper has preliminarily verified the application of the double-optimized AHNs method in the field of the IWM’s condition recognition. The real operating conditions of the EV are more complex, the speed changes frequently, and the duration is not constant, which greatly increases the application difficulty of the double-optimized AHNs method. Certainly, the interpretability of AHNs is still an issue. In the future, the double-optimized AHNs algorithm will be further optimized to lay a better foundation for the field of the on-line fault diagnosis of the IWM in the real environment. Moreover, the performances of the double-optimized AHNs with different parameters will be discussed in detail and compared with the advanced network models such as the CNN, deep belief network (DBN), and stacked auto-encoder network (SAN).

## Figures and Tables

**Table 1:** The average CPU ^1^ times of the three methods in the training and test processes.

Operating Condition		AHNs		K-AHNs		Proposed Method	
Speed [rpm]	Load [Nm]	Training [s]	Test [s]	Training [s]	Test [s]	Training [s]	Test [s]
1500	0.7	35.21	14.73	4.50	2.45	9.69	2.58
900	0.7	31.60	13.69	4.75	2.16	8.76	1.95
1500	0.1	35.47	14.74	4.56	2.10	8.89	2.13

^1^ Intel(R) Core(TM) i7-8700 CPU @ 3.20 GHz 3.19 GHz.

## Data Availability

Not applicable.
